# Prediction of One Repetition Maximum Using Reference Minimum Velocity Threshold Values in Young and Middle-Aged Resistance-Trained Males

**DOI:** 10.3390/bs11050071

**Published:** 2021-05-07

**Authors:** John F. T. Fernandes, Amelia F. Dingley, Amador Garcia-Ramos, Alejandro Perez-Castilla, James J. Tufano, Craig Twist

**Affiliations:** 1 Higher Education Sport, Hartpury University, Hartpury GL19 3BE, UK; amelia.dingley@hartpury.ac.uk; 2 Department of Physical Education and Sport, University of Granada, 18010 Granada, Spain; amagr@ugr.es (A.G.-R.); alexperez@ugr.es (A.P.-C.); 3 Department of Physical Education and Sport, Charles University, 110 00 Prague, Czech Republic; tufano@ftvs.cuni.cz; 4 Department of Sport and Exercise Sciences, University of Chester, Chester CH1 4BJ, UK; c.twist@chester.ac.uk

**Keywords:** aging, maximal strength, squat, bench press, bent-over-row, velocity-based training, linear position transducer

## Abstract

Background: This study determined the accuracy of different velocity-based methods when predicting one-repetition maximum (1RM) in young and middle-aged resistance-trained males. Methods: Two days after maximal strength testing, 20 young (age 21.0 ± 1.6 years) and 20 middle-aged (age 42.6 ± 6.7 years) resistance-trained males completed three repetitions of bench press, back squat, and bent-over-row at loads corresponding to 20–80% 1RM. Using reference minimum velocity threshold (MVT) values, the 1RM was estimated from the load-velocity relationships through multiple (20, 30, 40, 50, 60, 70, and 80% 1RM), two-point (20 and 80% 1RM), high-load (60 and 80% 1RM) and low-load (20 and 40% 1RM) methods for each group. Results: Despite most prediction methods demonstrating acceptable correlations (*r* = 0.55 to 0.96), the absolute errors for young and middle-aged groups were generally *moderate* to *high* for bench press (absolute errors = 8.2 to 14.2% and 8.6 to 20.4%, respectively) and bent-over-row (absolute error = 14.9 to 19.9% and 8.6 to 18.2%, respectively). For squats, the absolute errors were lower in the young group (5.7 to 13.4%) than the middle-aged group (13.2 to 17.0%) but still unacceptable. Conclusion: These findings suggest that reference MVTs cannot accurately predict the 1RM in these populations. Therefore, practitioners need to directly assess 1RM.

## 1. Introduction

The age-associated losses in muscle mass (i.e., sarcopenia) [1,2] and in strength and power (i.e., dynapenia) [3] contribute to age-related declines in athletic performance. Fortunately, resistance training is a potent strategy to improve muscle mass, power, and strength in aging populations [4,5]. Therefore, a growing number of middle-aged athletes are participating in resistance training not only to maintain or improve their athletic performance but also to slow down the natural age-associated declines in athletic performance [6].

For resistance training to be effective, it is important to quantify the stress imposed during training [7]. If the training load is insufficient then adaptation might not occur, whereas excessive or sudden increases in stress might result in injury, poor performance, or non-functional overreaching [8,9]. Therefore, although loading paradigms can be prescribed based upon a variety of methods, using evidence-based methods might help guide training with objective data, possibly increasing the effectiveness of training. Traditionally, resistance training loads have been prescribed based on a percentage of one-repetition maximum (direct method; %1RM). Although a properly structured training program using 1RM percent-based methods are effective at increasing muscle mass, strength, and performance, direct determination of 1RM is sometimes discouraged because repeated maximum lifts can be fatiguing, the procedure can be time-consuming, and one’s performance might vary from day-to-day [10]. Alternatively, auto-regulatory methods (e.g., repetitions in reserve) are gaining popularity but these are still subjective and may not be effective for all populations. At the opposite end of the spectrum from these subjective approaches, contemporary ‘velocity-based training’ methods might provide a more objective and precise prescription of resistance training intensities [11].

The inverse-linear relationship between the load lifted and movement velocity can be used to predict 1RM during various resistance training exercises [11]. Briefly, this load-velocity relationship can be modeled by assessing velocity over multiple submaximal loads (usually 3–8 loads; i.e., ‘multiple-point method’), with the 1RM being estimated via linear regression as the load associated with the velocity at 1RM (i.e., the minimum velocity threshold; MVT) [12,13,14]. This relationship can also be accurately modeled using two loads (i.e., ‘two-point method’) [11], though the loads and MVT selected in modeling need to be considered [15]. While previous work has investigated the ability of force- and velocity-biased loads when predicting maximum force and velocity, no study has compared the 1RM prediction accuracy from the two-point method, modeled by different load configurations (e.g., distant two-point, low-load method, and high-load method). When considering the MVT in the modeling, the low reliability of the individual MVT and the favorable between-participant variability, indicate that a reference MVT value for all participants could be used [11]. A study that identifies if maximal strength can be predicted using reference MVT values will provide practitioners with an easy, less fatiguing, and quick way of predicting 1RM in older populations.

Despite the literature emerging that velocity-based methods can be used to predict 1RM, its efficacy in older populations has received little attention. In old resistance-trained women (~68 years), Marcos-Pardo and colleagues [16] reported that mean velocity can accurately estimate the percentage 1RM (40 to 100%) in bench and leg press exercises. However, Marcos-Pardo et al. [16] did not include a younger group for comparison, so it remains unknown if the ability to predict the 1RM is affected by age. This is an important issue because the load-velocity relationship is flatter (i.e., more horizontal/less vertical) in middle-aged resistance-trained males than their young counterparts [17]. As such, it is plausible that the combination of loads to predict 1RM might be different in young and middle-aged populations. A study that determines the accuracy of 1RM predictions across resistance training exercises will aid coaches when prescribing training loads in middle-aged males. However, as no study has investigated this, we aimed to elucidate whether the load-velocity relationship can be used to estimate the 1RM across traditional resistance training exercises (such as bench press, back squat, and bent-over-row) in both middle-aged and young men. A secondary aim is to identify which configuration of the two-load method (i.e., distant two-point, high-load, and low-load) could predict 1RM most accurately in young and middle-aged resistance training males. Given the dearth of comparable studies, we propose the null hypothesis for both of our aims: (1) that there will be no systematic differences between actual and predicted 1RM in young and middle-aged men, and (2) the load combination selected will not affect the 1RM prediction accuracy.

## 2. Materials and Methods

### 2.1. Participants

Twenty young (age 21.0 ± 1.6 years, body mass 85.9 ± 12.8 kg) and 20 middle-aged (age 42.6 ± 6.7 years, body mass 82.3 ± 11.2 kg) males were recruited by convenience sampling. The lower- (35 years) and upper-age (55 years) limits were selected for the older group in line with previous studies [3,17,18]. All participants regularly used the bench press, back squat, and bent-over-row as part of their training programs and had a minimum of 2 years resistance training experience (4.5 ± 1.1 and 16.9 ± 11.4 years for young and middle-aged groups, respectively). A sample size of 38 persons (19 per group) was estimated using G*power based upon an effect size, alpha error probability, and power of 1.1 (as observed between groups [19] for handgrip power at 50% maximal voluntary contraction), 0.05 and 0.95, respectively. Participants completed a pre-test health questionnaire and provided written consent for the study, which was approved by the host institution’s faculty ethics committee (871/14/JF/SES). Participants were instructed not to consume any ergogenic supplements (for example, caffeine) on the day of testing, and to refrain from strenuous exercise in the 3 h before testing. If it did not conflict with the current study, participants continued their normal training programs. Moreover, discussions with participants before testing revealed no symptoms of perceived muscle soreness or weakness.

### 2.2. Study Design

This study comprised a mixed factorial design in which two groups of participants attended the laboratory twice. On the first visit, maximal strength was assessed for the bench press, back squat, and bent-over-row, followed by habituation to the measures of barbell mean velocity (i.e., until the mean velocity plateaued). Participants returned to the laboratory 48 h later to complete 3 repetitions of the bench press, back squat, and bent-over-row at loads corresponding to 20 to 80% 1RM (at 10% 1RM increments) in a randomized order for both exercises and loads. Mean velocity (i.e., the average velocity across the concentric phase) of all the repetitions performed at each relative load was recorded by a linear position transducer. Thereafter, four velocity-based methods were used to predict 1RM; the data of seven loads (20, 30, 40, 50, 60, 70, and 80% 1RM; i.e., multiple-load method), two distant loads (20 and 80% 1RM; i.e., distant two-point method), two light loads (20 and 40% 1RM; i.e., low method), and two heavy loads (60 and 80% 1RM; i.e., high-load method) were used to model the individual load-velocity relationships by linear regression. The 1RM was estimated from the load-velocity relationship as the load associated with reference MVT values of 0.17 m·s^−1^ for bench press [10,15], 0.37 m·s^−1^ for back squat [20], and 0.40 m·s^−1^ for bent-over-row [21].

### 2.3. Maximal Strength Testing (Session 1)

Bench press and bent-over-row maximal strength were directly assessed using a standard 1RM protocol [22]. For safety and ethical reasons, the 1RM back squat was predicted from a 5RM protocol and equation detailed previously (*R*^2^ = 0.988) [23]. A similar approach has been used for back squat exercise [24]. The exercise order was randomized with 5 min passive rest between 1RMs. All exercises were performed on the Smith machine (Smith machine standard; Perform Better, Leicester, UK).

### 2.4. Assessment of Mean Velocity (Session 2)

Mean velocity was assessed during the three exercises at loads corresponding to 20, 30, 40, 50, 60, 70, and 80% 1RM. Loads were applied randomly, and the FitroDyne rotary encoder (Fitronic, Bratislava, Slovakia) was attached directly under and to the most lateral aspect of the Smith machine bar. Since the FitroDyne measures, the rate of displacement and thus assumes that the nylon cord is moving in a vertical direction, any deviation from the vertical direction could increase measurement error. As such, the Smith machine was employed as it restricts the movement of the nylon cord to a linear vertical direction. The FitroDyne provides a reliable marker of moderate changes in mean velocity for the selected loads and exercises [25].

For the bench press exercise, the participants held the bar with a prone grip and lowered it to their chest, before maximally pushing it until full elbow extension. For the back squat exercise, the bar was positioned in a high-bar position and the participants descended until their hips were below the knee joint, and then ascended as rapidly as possible until their knees were at full extension. An adjustable bench was positioned at the bottom of the back squat to signal the eccentric-to-concentric transition, ensuring that each participant attained the same depth and range of motion on each repetition. During the bent-over-row exercise, the participants commenced in a bent-over position (i.e., back angle of approximately 45 degrees), before pulling the bar maximally until the elbows reached full flexion (no eccentric phase). For all exercises, participants were instructed to perform the eccentric phase in a controlled manner and the concentric phase as rapidly as possible. Three repetitions of each exercise were performed at each load with self-selected rest intervals that were capped at 90 s, but ranged from 30 to 90 s. Rest times were self-selected as lighter loads did not require the same recovery time. The average of the three repetitions at each load was used to model the load-velocity relationships.

### 2.5. Statistical Analysis

A two-way analysis of variance (ANOVA), with a group (young and middle-aged) as a between-subject factor and the 1RM prediction method (multiple-point, distant two-point, high-load, and low-load) as a within-subject factor, was applied to the absolute differences between the actual and predicted 1RMs separately for each exercise. The absolute differences were expressed as a percentage of the actual 1RM because the differences in the 1RM strength between young and middle-aged men could affect the between-group comparisons. The Greenhouse-Geisser correction was applied when the assumption of the homogeneity of the variances was violated (*p* < 0.05). Paired samples *t*-tests with Bonferroni *post hoc* corrections were used to identify bias between specific pairwise comparisons. The scale used to categorize the magnitude of the absolute errors was: *low* (<5.0%), *moderate* (5.0–10%), and *high* (>10.0%) [26]. Furthermore, the validity of the 1RM prediction methods with respect to the actual 1RM was examined through paired samples *t*-tests, Cohen’s *d* effect size (ES), raw differences (±standard deviation), and the heteroscedasticity of the errors (*r*; the relationship of the raw differences between the actual and predicted 1RMs with their average value). Although not indicative of agreement [27], Pearson’s correlation coefficients (*r*) were calculated to facilitate the comparisons to previous research. Qualitative interpretations of the *r* coefficients were defined as follows *trivial* (0.00–0.09), *small* (0.10–0.29), *moderate* (0.30–0.49), *large* (0.50–0.69), *very large* (0.70–0.89), *nearly perfect* (0.90–0.99), and *perfect* (1.00) [28]. The magnitude of the ES was interpreted as follows: *trivial* (<0.20), *small* (0.20–0.59), *moderate* (0.60–1.19), *large* (1.20–2.00), and *very large* (>2.00) [28]. Heteroscedasticity of error was defined as an *r* > 0.32 [29]. Alpha was set at 0.05. All data were calculated using SPSS software (version 26, IBM SPSS, INC., Chicago, IL, USA). 

## 3. Results

### 3.1. Bench Press

**Figure 1:**
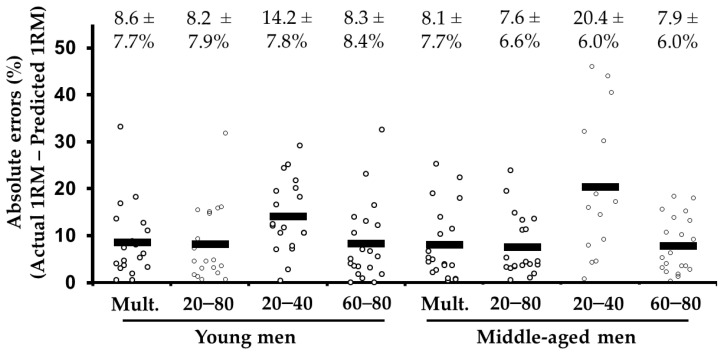
Comparison of the absolute differences (mean ± SD), expressed as a percentage, between the actual 1 repetition maximum (1RM) and the 1RM estimated from the different prediction methods in the bench press exercise. Note the black rectangles represent the median values.

Seven out of 160 predicted 1RMs were considered outliers (i.e., the absolute errors were >50%; 1 young and 6 middle-aged males for the low-load method) and were not considered for statistical analyses. The ANOVA revealed differences in the 1RM prediction method (*F*_(1.3, 41.6)_ = 11.6, *p* = 0.001), with higher errors for the low-load method compared to the multiple-point (*p* = 0.004), distant two-point (*p* = 0.002), and high-load (*p* = 0.018) methods. The magnitude of the absolute errors was similar between the multiple-point, distant two-point, and high-load methods (all *p* = 1.00). The main effect of group (*F*_(1, 31)_ = 0.49, *p* = 0.488) and the 1RM prediction method by group interaction (*F*_(1.3, 41.6)_ = 1.5, *p* = 0.232) effect did not reach significance. The errors were *moderate* for both young and middle-aged males using the multiple-point, distant two-point, and low-load predictions (range: 7.6% to 8.6%) and *high* for the low-load method (14.2 and 20.4% in the young and middle-aged groups, respectively; Figure 1).

*Trivial* differences (raw differences range: −2.1 to 3.5 kg), *very large* to *nearly perfect* correlations (*r* range: 0.79 to 0.90), and homoscedastic errors (*r* range: −0.01 to 0.21) were observed between the actual 1RM and the 1RMs predicted by the multiple-point, distant two-point, and high-load methods in the groups of young and middle-aged groups (Table 1). Higher raw differences (from −0.7 to 11.0 kg), *small* to *moderate* correlations (*r* range: 0.58 to 0.63), and heteroscedastic errors (*r* range: 0.31 to 0.68) were observed for the low-load method in the whole and middle-aged groups. 

### 3.2. Back Squat

**Figure 2:**
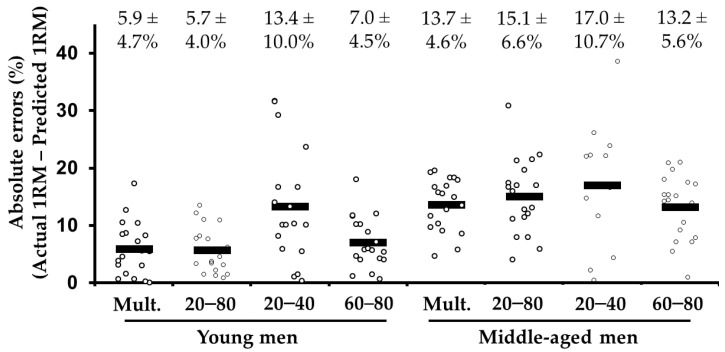
Comparison of the absolute differences (mean ± SD), expressed as a percentage, between the actual 1 repetition maximum (1RM) and the 1RM estimated from the different prediction methods in the back squat exercise. Note the black rectangles represent the median values.

Two subjects (1 young and 1 middle-aged male) did not complete the back squat protocol due to discomfort. Seven out of 152 predicted 1RMs were considered outliers (i.e., the absolute errors were >50%; 1 young and 6 middle-aged men for the low-load method) and were not considered for statistical analyses. The ANOVA revealed a main effect of the 1RM prediction method (*F*_(1.5, 42.2)_ = 6.9, *p* = 0.006) with higher errors for the low-load compared to the multiple-point (*p* = 0.013), distant two-point (*p* = 0.063), and high-load (*p* = 0.096) methods. The magnitude of the absolute errors between the multiple-point, distant two-point, and high-load methods was comparable (all *p* = 1.00). There was also a main effect of group was also significant (*F*_(1, 29)_ = 14.4, *p* = 0.001) due to higher error for middle-aged (range = −1.7 to −7.2 kg) compared to young group (range = −0.9 to 1.1 kg). The interaction between 1RM prediction method and group did not reach statistical significance (*F*_(1.5, 42.2)_ = 0.8, *p* = 0.418). The errors were *moderate* for the young group using the multiple-point, distant two-point, and high-load methods (range: 5.7% to 7.0%), but *high* errors were always obtained for the middle-aged group and using the low-load method (Figure 2).

Generally, *trivial* differences were observed between the actual and predicted 1RMs (*p* range: 0.071 to 0.965) with the only exception being the high-load method in the middle-aged group that overestimated the 1RM by 7.2 kg (*p* = 0.037; Table 2). The correlations between the actual 1RM and the 1RM estimated using the multiple-point, distant two-point, and high-load methods were *nearly perfect* for the young group (*r* range: 0.95 to 0.96) and *very large* for the middle-aged group (*r* range: 0.73 to 0.81). Weaker correlations (*r* range: 0.62 to 0.74) were obtained using the low-load method. Heteroscedasticity of errors (*r* range: 0.36 to 0.68) were observed for the young group, with higher 1RM values being associated with higher differences in favor of the predicted 1RMs, while no heteroscedasticity of errors (*r* range: 0.21 to 0.59) was generally observed for the middle-aged group.

### 3.3. Bent-over-Row

**Figure 3:**
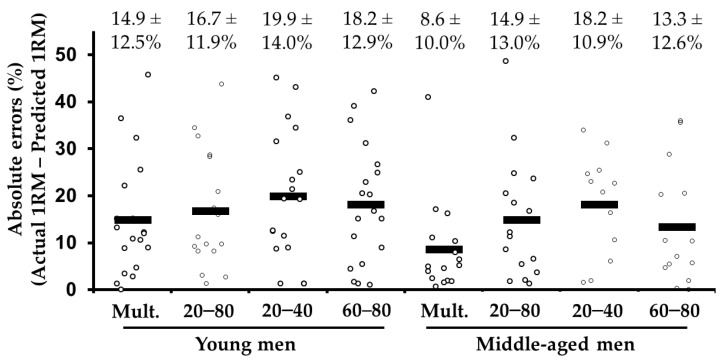
Comparison of the absolute differences (mean ± SD), expressed as a percentage, between the actual 1 repetition maximum (1RM) and the 1RM estimated from the different prediction methods in the bent-over-row exercise. Note the black rectangles represent the median values.

Data of 5 subjects (1 young and 4 middle-aged males) were excluded for all 1RM prediction methods due to systematic absolute errors above 50%. From the remaining 140 predicted 1RMs, we considered 5 outliers using the low-load (1 young and 4 middle-aged males) and 2 using the high-load (both middle-aged males) methods. The main effect of the 1RM prediction method (*F*_(1.9, 48.7)_ = 3.0, *p* = 0.61), group (*F*_(1, 26)_ = 2.9, *p* = 0.099), and the interaction between 1RM prediction method and group (*F*_(1.9, 48.7)_ = 0.6, *p* = 0.602) were similar (Figure 3). The errors were always *high* (range: 13.3% to 19.9%) with the only exception being the multiple-point for the middle-aged group, which showed *moderate* errors (8.6%) (Figure 3).

The multiple-point, distant two-point and high-load methods overestimated the actual 1RM the correlations were *very large,* and the errors were generally heteroscedastic with greater 1RM values being associated with higher differences in favor of the predicted 1RMs (*r* range: 0.31 to 0.71) (Table 3). *Trivial* to *small* differences were noted between the 1RM predicted using the low-load method and the actual 1RM, but the correlations were weaker than the other methods (*r* range: 0.55 to 0.72), and the errors were still heteroscedastic (*r* range: 0.47 to 0.74).

## 4. Discussion

This is the first study to compare the accuracy of the load-velocity relationship in predicting maximal strength between young and middle-aged resistance-trained males. Regardless of the exercise, the low-load method presented the lowest accuracy in the prediction of the 1RM. The multiple-point, distant two-point, and high-load methods, across all exercises, presented similar inaccuracy with respect to the actual 1RM. The main findings of the study were that the errors in the prediction of the 1RM were comparable for the young and middle-aged group in the bench press (moderate errors) and bent-over-row (high errors), while the middle-aged group presented greater errors than their young counterparts in the back squat exercise (high versus moderate errors, respectively). These results suggest that the load-velocity relationship should not be used to predict the 1RM in young and middle-aged males during bench press, back squat, and bent-over-row exercises when using reference MVT values. 

Similar to previous reports, the current data demonstrated a *very* large *to* a *nearly perfect* relative agreement with respect to the actual 1RM and predicted 1RM using multiple-point and distant two-point methods for bench press exercise [10,30,31]. However, measures of association are not indicative of the absolute agreement between 1RM prediction methods; more important is the absolute error which in this study is *moderate* and, in the case of the velocity-biased two-load method, *high*. These absolute errors are alarming for those who use such methods in practice and are in contrast to previous reports of a favorable agreement between direct 1RM assessment and velocity-based 1RM prediction methods in the bench press exercise [10,31,32]. The absolute errors, which were similar between groups, demonstrate variability which challenges the ability of these 1RM prediction methods in detecting changes in upper-body pushing strength (~9.1 kg) observed after resistance training in older adults [33]. Moreover, if different 1RM predictions methods are used in-season, these might infer a change has occurred despite observations of no change in bench press strength across this period [34]. Therefore, we suggest that practitioners do not assess bench press maximal strength using these velocity-based methods in young and middle- resistance-trained males.

The mostly *very large* and *nearly perfect* correlations between prediction methods for the back squat were coupled with absolute errors that were large in the middle-aged group than their young counterparts. These data suggest that 1RM cannot be predicted from the load-velocity relationship in the back squat exercise, which reaffirms previous studies [12,13]. Our errors were substantially smaller than those observed by Hughes et al. [13], likely owing to the different statistics used in their study and ours (95% limits of agreement and absolute and relative differences, respectively). Similarly, Banyard et al. [12] observed poor agreement when the 1RM was predicted from 5 (7.4% coefficient of variation; CV), 4 (9.1% CV), and 3 (12.8% CV) loads. Nonetheless, the errors here were larger for the middle-aged groups (range = 13.2 to 17.0%) than the younger group (range = 5.7 to 13.4%). It is unclear why these errors were higher for the middle-aged group, but it might be owing to the greater dynapenia that occurs in the lower-body than upper-body [35,36] and the subsequent effect on the slope/shape of the load-velocity relationship [17,37]. The reasons for this aggravated dynapenia is likely owing to the reduced usage of the lower-body with age [38] and more severe changes in contractile units (e.g., decreases in the specific tension of types 1 and 2 fibers) [39] and connective tissues (e.g., increases in fat and connective tissue) [40]. Nonetheless, such large errors would *theoretically* be able to detect large increases in lower-body strength that have been observed in some studies (e.g., ~42.1 kg, Conlon et al., 2016) but the errors in both groups are probably too large to detect the small increases (~5–10%) that are recommended when adjusting load within a micro-cycle [12,41]. Similarly, the improvements in back squat 1RM that occur in-season are typically small (e.g., ~8.3 kg, Hoffman & Kang, 2003), which challenges the ability of the velocity-based 1RM prediction methods used in the present study to detect these changes. Readers should be aware that for safety and ethical reasons, maximal strength was calculated from a 5RM and not assessed via a direct 1RM. Therefore, some values 1RM values for the back squat could be over or underestimated. Nonetheless, our data suggest that these velocity-based methods cannot be used to predict the back squat 1RM in young and middle-aged resistance-trained males.

For the bent-over-row exercise, our findings reflect a poor level of agreement between 1RM and the velocity-based 1RM predicted methods. Independent of group and prediction method, the *very large* correlations were coupled with unfavorable absolute errors (range = 8.6 to 19.9%). The feasibility of the load-velocity relationship when predicting 1RM in upper-body pulling exercises has been investigated before. In both the bent-over-row [42], seated-cable row [21] and prone-bench pull [43] exercises, the load-velocity relationship was able to predict the 1RM (random errors = ~3.1, 3.5, and 5.4 kg, respectively). The poor agreement observed in our study is likely due to the use of a reference MVT value rather than those specific to that sample [21,42,43]. Moreover, the MVT used by Loturco and colleagues [42] ~0.630 m·s^−1^ was similar to the mean velocity at 80% 1RM in the current study (~0.638 m·s^−1^). The reasons for this are unclear, especially given that our 1RM values (90.2 ± 14.8 kg) are comparable to those of the athletes in Loturco et al.’s [42] work (88.5 ± 13.1 kg). We selected a reference MVT value used during a similar pulling exercise, the seated cable row [21], which given the overestimations, *might* suggest a slower reference MVT value is needed. Irrespective of the differences between these studies, the absolute errors in the current work challenge the ability of these predictive methods to detect improvements in bent-over-row that have occurred after 6 [44] to 7 weeks [45] of resistance training (~5.1 and 15 kg, respectively). Like the bench press and back squat errors, these are likely too large to detect the small increases in load that occur across micro-cycles or between sessions within a microcycle [12,41]. As with bench press and back squat, we do not recommend using these prediction methods to estimate bent-over-row 1RM in young and middle-aged resistance trained males.

The poor levels of agreement between the 1RM assessment methods reported here are undesirable from the perspective of detecting meaningful changes in strength or prescribing training loads. Primarily, the errors in this study could be inflated due to day-to-day variability in strength. In this study, the actual 1RM and the load-velocity relationship were assessed in different sessions unlike previous studies (e.g., [21,43]) in which lower errors were reported when the 1RM and load-velocity relationships were evaluated in the same session. Future studies should also elucidate whether the higher errors reported in this study could be caused by the loads being administered randomly rather than in incremental order. Previous work in isokinetic dynamometry has suggested that slow angular velocities should be applied before faster angular velocities [46], but whether this is the case in isoinertial exercise remains unknown. Secondly, the poor agreement might be owing to the use of reference MVT values, instead of those specific to the sample in the study. The particularly poor agreement in the bent-over-row, where the prediction methods typically overestimated 1RM, would suggest that the use of a lower MVT could increase the accuracy of the prediction. A final explanation might be owing to the measurement tool. Though the FitroDyne is reliable [25], different linear position transducers, even those working via similar technologies, do not always provide comparable data [27,32]. Previously, Fernandes and colleagues [27] observed differences of ± 0.04 to 0.12 m·s^−1^ between two commonly used linear position transducers during bent-over-row exercise. Moreover, the MVT observed by Loturco et al. [42] is comparable to the mean velocity at 80% 1RM in the current study. It is unlikely that this is wholly explained by differences in samples, given their similar characteristics, but rather the different tools might account for *some* of the discrepancies. This reaffirms the need for practitioners to attain an MVT value that is specific to their measurement tool, specific to each exercise, and to each individual. 

Readers should be aware of the several outliers (i.e., absolute errors >50%) removed from the data analysis. Predominantly, these outliers were found in the middle-aged group using the low-load prediction method. A manual inspection of these outliers showed that the differences in velocity between 20 and 40% 1RM were small (i.e., the slope was flatter than between other load configurations). This suggests that this population, although well resistance-trained, is not accustomed to maximal intent with low loads. Additionally, the aging-associated slowing of the muscle [17] changes in fascicle length [47], reduced ATPase activity [39], and changes in contractile properties (i.e., increased slow myosin heavy chain content) [48] which contribute to maximal velocity contractions, contributed to flattening of the slope between 20 and 40% 1RM. It is also plausible that the FitroDyne does not possess sufficient reliability to determine the movement velocity at these loads. Nonetheless, that several outliers had to be removed, further reinforces that reference MVT values should not be used to predict 1RM in middle-aged resistance-trained males.

## 5. Conclusions

These data indicate that the 1RM cannot be accurately predicted from the load-velocity relationship in young and middle-aged males when using reference MVT values. Moreover, the load combination used did not influence the prediction of maximal strength from the load-velocity relationship. The poor agreement between velocity-based methods observed in this study was independent of the groups for bench press and bent-over-row, suggesting that the age-related differences do not influence the prediction of 1RM from the load-velocity relationship. However, for the back squat, these prediction methods demonstrate a higher error in the middle-aged than the young group, which we attribute to age-associated changes in the shape of the load-velocity relationship caused by dynapenia. Practically, we do not recommend using reference MVT values to predict 1RM in these populations. Instead, if practitioners want to implement a 1RM mean velocity prediction value, it should be specific to their population, training situation, and device.

## Figures and Tables

**Table 1:** Differences, associations, and heteroscedasticity of the errors between the actual and predicted 1RMs during the bench press exercise.

Group	1RM Prediction Method	Raw Diff (kg)	*p*-Value	ES	*r* _(*Pearson*)_	*r* _(*heteroscedasticity*)_
Whole	Multiple-point (*n* = 40)	−0.4 ± 10.4	0.815	−0.02	0.85	0.00
	Distant two-point (*n* = 40)	1.0 ± 9.6	0.531	0.05	0.87	−0.01
	Low-load (*n* = 33)	4.3 ± 19.6	0.220	0.21	0.58	0.38 ^
	High-load (*n* = 40)	1.6 ± 9.9	0.324	0.08	0.87	0.09
Young	Multiple-point (*n* = 20)	−2.1 ± 11.8	0.438	−0.12	0.79	0.13
	Distant two-point (*n* = 20)	−0.9 ± 11.4	0.743	−0.05	0.80	0.14
	Low-load (*n* = 19)	−0.7 ± 17.7	0.870	−0.03	0.63	0.31
	High-load (*n* = 20)	−0.3 ± 11.7	0.902	−0.02	0.81	0.21
Middle-aged	Multiple-point (*n* = 20)	1.3 ± 8.7	0.504	0.08	0.86	0.04
	Distant two-point (*n* =20)	2.8 ± 7.1	0.098	0.17	0.90	0.02
	Load-load (*n* = 14)	11.0 ± 20.7	0.069	0.56	0.61	0.68 ^
	High-load (*n* = 20)	3.5 ± 7.6	0.056	0.20	0.90	0.21

Data are mean ± standard deviation. Raw diff, Raw differences; ES, Cohen’s *d* effect size ([Predicted 1RM—Actual 1RM]/SD both); *r_Pearson_*, Pearson’s correlation coefficient; *r_heteroscedasticity_*, heteroscedasticity of the errors; ^ denotes heteroscedasticity (i.e., *r* > 0.32).

**Table 2:** Differences, associations, and heteroscedasticity of the errors between the actual and predicted 1RMs during the back squat exercise.

Group	1RM Prediction Method	Raw Diff (kg)	*p*-Value	ES	*r* _(*Pearson*)_	*r* _(*heteroscedasticity*)_
Whole	Multiple-point (*n* = 38)	−1.3 ± 18.8	0.570	−0.04	0.92	0.45 ^
	Distant two-point (*n* = 38)	−1.7 ± 14.4	0.463	−0.05	0.91	0.42 ^
	Load-load (*n* = 31)	−1.0 ± 24.1	0.814	−0.03	0.74	0.39 ^
	High-load (*n* = 38)	−4.0 ± 13.4	0.071	−0.13	0.93	0.48 ^
Young	Multiple-point (*n* = 19)	1.1 ± 12.1	0.689	0.04	0.95	0.66 ^
	Distant two-point (*n* = 19)	0.1 ± 10.8	0.965	0.00	0.96	0.68 ^
	Load-load (*n* = 18)	−0.5 ± 24.9	0.927	−0.02	0.71	0.36 ^
	High-load (*n* = 19)	−0.9 ± 12.5	0.755	−0.03	0.95	0.65 ^
Middle-aged	Multiple-point (*n* = 19)	−3.7 ± 15.3	0.306	−0.16	0.78	0.21
	Distant two-point (*n* =19)	−3.6 ± 17.4	0.382	−0.16	0.73	0.26
	Load-load (*n* = 13)	−1.7 ± 23.8	0.802	−0.07	0.62	0.59 ^
	High-load (*n* = 19)	−7.2 ± 13.9	0.037 *	−0.32	0.81	0.18

Data are mean ± standard deviation. Raw diff, Raw differences; ES, Cohen’s *d* effect size ([Predicted 1RM—Actual 1RM]/SD both); *r_Pearson_*, Pearson’s correlation coefficient; *r_heteroscedasticity_*, heteroscedasticity of the errors; *, *p* is < 0.05; ^ denotes heteroscedasticity (i.e., *r* > 0.32).

**Table 3:** Differences, associations, and heteroscedasticity of the errors between the actual and predicted 1RMs during the bent-over-row exercise.

Group	1RM Prediction Method	Raw Diff (kg)	*p*-Value	ES	*r* _(*Pearson*)_	*r* _(*heteroscedasticity*)_
Whole	Multiple-point (*n* = 35)	6.4 ± 14.1	0.011 *	0.34	0.77	0.50 ^
	Distant two-point (*n* = 35)	10.3 ± 14.5	<0.001 *	0.56	0.74	0.44 ^
	Load-load (*n* = 30)	−2.4 ± 20.8	0.403	−0.14	0.72	0.68 ^
	High-load (*n* = 33)	10.1 ± 17.0	0.002 *	0.50	0.77	0.66 ^
Young	Multiple-point (*n* = 19)	8.3 ± 16.9	0.046 *	0.43	0.71	0.56 ^
	Distant two-point (*n* = 19)	10.4 ± 16.6	0.014 *	0.54	0.71	0.54 ^
	Load-load (*n* = 18)	0.3 ± 23.6	0.964	0.01	0.68	0.74 ^
	High-load (*n* = 19)	10.9 ± 19.1	0.023 *	0.52	0.72	0.66 ^
Middle-aged	Multiple-point (*n* = 16)	4.2 ± 9.9	0.110	0.29	0.78	0.31
	Distant two-point (*n* = 16)	10.3 ±12.1	0.004 *	0.69	0.70	0.35 ^
	Load-load (*n* = 12)	−8.4 ± 15.2	0.080	−0.56	0.55	0.47 ^
	High-load (*n* = 14)	9.0 ± 14.4	0.035 *	0.50	0.81	0.71 ^

Data are mean ± standard deviation. Raw diff, Raw differences; ES, Cohen’s *d* effect size ([Predicted 1RM—Actual 1RM]/SD both); *r_Pearson_*, Pearson’s correlation coefficient; *r_heteroscedasticity_*, heteroscedasticity of the errors; *, *p* is < 0.05; ^ denotes hetereoscedasticity (i.e., *r* > 0.32).

## Data Availability

Not applicable.

## References

[1] Lexell J., Taylor C.C., Sjöström M. (1988). What is the cause of the ageing atrophy? Total number, size and proportion of different fiber types studied in whole vastus lateralis muscle from 15- to 83-year-old men. J. Neurol. Sci..

[2] Fernandes J.F.T., Lamb K.L., Norris J.P., Moran J., Drury B., Borges N.R., Twist C. (2020). Aging and recovery after resistance-exercise-induced muscle damage: Current evidence and implications for future research. J. Aging Phys. Act..

[3] Fernandes J.F.T., Lamb K.L., Twist C. (2019). Exercise-induced muscle damage and recovery in young and middle-aged males with different resistance training experience. Sports.

[4] Newton R.U., Häkikinen K., Häkikinen A., Mccormick M., Volek J., Kraemer W. (2002). Mixed-methods of resistance training increases power and strength of young and older men. Med. Sci. Sports Exerc..

[5] Roth S.M., Martel G.F., Ivey F.M., Lemmer J.T., Tracy B.L., Hurlbut D.E., Metter E.J., Hurley B.F., Rogers M.A. (1999). Ultrastructural muscle damage in young vs. older men after high-volume, heavy-resistance strength training. J. Appl. Physiol..

[6] Tanaka H., Seals D.R. (2008). Endurance exercise performance in Masters athletes: Age-associated changes and underlying physiological mechanisms. J. Physiol..

[7] Scott B.R., Duthie G.M., Thornton H.R., Dascombe B.J. (2016). Training monitoring for resistance exercise: Theory and applications. Sports Med..

[8] Halson S.L. (2014). Monitoring training load to understand fatigue in athletes. Sports Med..

[9] Bell L., Ruddock A., Maden-Wilkinson T., Rogerson D. Overreaching and overtraining in strength sports and resistance training: A scoping review. J. Sports Sci..

[10] García-Ramos A., Haff G.G., Pestaña-Melero F.L., Pérez-Castilla A., Rojas F.J., Balsalobre-Fernández C., Jaric S. (2018). Feasibility of the 2-point method for determining the 1-repetition maximum in the bench press exercise. Int. J. Sports Physiol. Perform..

[11] Weakley J., Mann B., Banyard H., Mclaren S., Scott T., Garcia-ramos A. (2020). Velocity-based training: From theory to application. Strength Cond. J..

[12] Banyard H.G., Nosaka K., Haff G.G. (2017). Reliability and validity of the load-velocity relationship to predict the 1RM back squat. J. Strength Cond. Res..

[13] Hughes L.J., Banyard H.G., Dempsey A.R., Scott B.R. (2019). Using a load-velocity relationship to predict one repetition maximum in free-weight exercise: A comparison of the different methods. J. Strength Cond. Res..

[14] Caven E.J.G., Bryan T.J.E., Dingley A.F., Drury B., Garcia-Ramos A., Perez-Castilla A., Arede J., Fernandes J.F.T. (2020). Group versus individualised minimum velocity thresholds in the prediction of maximal strength in trained female athletes. Int. J. Environ. Res. Public Health.

[15] Perez-Castilla A., Fernandes J.F.T., Garcia-Ramos A. (2021). Validity of the bench press one-repetition maximum test predicted through individualized load-velocity relationship using different repetition criteria and minimal velocity thresholds. Isokinet. Exerc. Sci..

[16] Marcos-Pardo P.J., González-Hernández J.M., García-Ramos A., López-Vivancos A., Jiménez-Reyes P. (2019). Movement velocity can be used to estimate the relative load during the bench press and leg press exercises in older women. PeerJ.

[17] Fernandes J.F.T., Lamb K.L., Twist C. (2018). A comparison of load-velocity and load-power relationships between well-trained young and middle-aged males during three popular resistance exercises. J. Strength Cond. Res..

[18] Fernandes J.F.T., Lamb K.L., Twist C. (2018). Internal loads, but not external loads and fatigue, are similar in young and middle-aged resistance-trained males during high volume squatting exercise. J. Funct. Morphol. Kinesiol..

[19] Demura S., Aoki H., Sugiura H. (2011). Gender differences in hand grip power in the elderly. Arch. Gerontol. Geriatr..

[20] Pallarés J.G., Sánchez-Medina L., Pérez C.E., De La Cruz-Sánchez E., Mora-Rodriguez R. (2014). Imposing a pause between the eccentric and concentric phases increases the reliability of isoinertial strength assessments. J. Sports Sci..

[21] Perez-Castilla A., Suzovic D., Domanovic A., Fernandes J.F.T., Garcia-Ramos A. Validity of different velocity-based methods and repetitions-to-failure equations for predicting the 1 repetition maximum during 2 upper-body pulling exercises. J. Strength Cond. Res..

[22] Stock M., Beck T.W., DeFreitas J., Dillon M. (2011). Test-retest reliability of barbell velocity during the free-weight bench-pres exercise. J. Strenght Cond. Res..

[23] Reynolds J., Gordon T., Robergs R. (2006). Prediction of one repetition maximum strength from multiple repetition maximum testing and anthropometry. J. Strength Cond. Res..

[24] Bazuelo-Ruiz B., Padial P., García-Ramos A., Morales-Artacho A.J., Miranda M.T., Feriche B. (2015). Predicting maximal dynamic strength from the load-velocity relationship in squat exercise. J. Strength Cond. Res..

[25] Fernandes J.F.T., Lamb K.L., Twist C. (2016). The intra- and inter-day reproducibility of the FitroDyne as a measure of multi-jointed muscle function. Isokinet. Exerc. Sci..

[26] Garcia-Ramos A., Jukic I., Weakley J., Janicijevic D. Bench press one-repetition maximum estimation through the individualised load-velocity relationship: Comparison of different regression models and minimal velocity thresholds. Int. J. Sports Physiol. Perform..

[27] Fernandes J.F.T., Lamb K.L., Clark C.C.T., Moran J., Drury B., Garcia-Ramos A., Twist C. Comparison of the FitroDyne and GymAware rotary encoders for quantifying peak and mean velocity during traditional multijointed exercises. J. Strength Cond. Res..

[28] Hopkins W.G., Marshall S.W., Batterham A.M., Hanin J. (2009). Progressive statistics for studies in sports medicine and exercise science. Med. Sci. Sports Exerc..

[29] Atkinson G., Nevill A.M. (1998). Statistical methods for assessing measurement error (reliability) in variables relevant to sports medicine. Sports Med..

[30] Pérez-Castilla A., Piepoli A., Garrido-Blanca G., Delgado-García G., Balsalobre-Fernández C., García-Ramos A. (2019). Precision of 7 commercially available devices for predicting bench-press 1-repetition maximum from the individual load–velocity relationship. Int. J. Sports Physiol. Perform..

[31] Pérez-Castilla A., Jerez-Mayorga D., Martínez-García D., Rodríguez-Perea Á., Chirosa-Ríos L.J., García-Ramos A. (2020). Comparison of the bench press one-repetition maximum obtained by different procedures: Direct assessment vs. lifts-to-failure equations vs. two-point method. Int. J. Sports Sci. Coach..

[32] Pérez-Castilla A., Piepoli A., Delgado-García G., Garrido-Blanca G., García-Ramos A. (2019). Reliability and concurrent validity of seven commercially available devices for the assessment of movement velocity at different intensities during the bench press. J. Strength Cond. Res..

[33] Conlon J.A., Newton R.U., Tufano J.J., Banyard H.G., Hopper A.J., Ridge A.J., Haff G.G. (2016). Periodization strategies in older adults: Impact on physical function and health. Med. Sci. Sports Exerc..

[34] Hoffman J.R., Kang J. (2003). Strength changes during an in-season resistance-training program for football. J. Strength Cond. Res..

[35] Candow D.G., Chilibeck P.D. (2005). Differences in size, strength, and power of upper and lower body muscle groups in young and older men. J. Gerontol. Biol. Sci..

[36] Frontera W.R., Suh D., Krivickas L.S., Hughes V.A., Goldstein R., Roubenoff R. (2000). Skeletal muscle fiber quality in older men and women. Am. J. Physiol. Cell Physiol..

[37] Raj I.S., Bird S.R., Shield A.J. (2010). Aging and the force-velocity relationship of muscles. Exp. Gerontol..

[38] Macaluso A., De Vito G. (2004). Muscle strength, power and adaptations to resistance training in older people. Eur. J. Appl. Physiol..

[39] Larsson L., Li X., Frontera W.R. (1997). Effects of aging on shortening velocity and myosin isoform composition in single human skeletal muscle cells. Am. J. Physiol..

[40] Lynch N.A., Metter E.J., Lindle R.S., Fozard J.L., Tobin J.D., Roy T.A., Fleg J.L., Hurley B.F. (1999). Muscle quality. I. Age-associated differences between arm and leg muscle groups. J. Appl. Physiol..

[41] Bompa T., Haff G.G. (2017). Periodization: Theory and Methodology of Training.

[42] Loturco I., Suchomel T., Kobal R., Arruda A.F.S., Guerriero A., Pereira L.A., Pai C.N. (2018). Force-velocity relationship in three different variations of prone row exercises. J. Strength Cond. Res..

[43] García-Ramos A., Barboza-González P., Ulloa-Díaz D., Rodriguez-Perea A., Martinez-Garcia D., Guede-Rojas F., Hinojosa-Riveros H., Chirosa-Ríos L.J., Cuevas-Aburto J., Janicijevic D. (2019). Reliability and validity of different methods of estimating the one-repetition maximum during the free-weight prone bench pull exercise. J. Sports Sci..

[44] Ratamess N.A., Beller N.A., Gonzalez A.M., Spatz G.E., Hoffman J.R., Ross R.E., Faigenbaum A.D., Kang J. (2016). The effects of multiple-joint isokinetic resistance training on maximal isokinetic and dynamic muscle strength and local muscular endurance. J. Sports Sci. Med..

[45] Samson A., Pillai P.S. (2018). Effect of cluster training versus traditional training on muscular strength among recreationally active males - A comparative study. Indian J. Physiother. Occup. Ther. Int. J..

[46] Wilhite M.R., Cohen E.R., Wilhite S.C. (1992). Reliability of concentric and eccentric measurements of quadriceps performance using the KIN-COM dynamometer: The effect of testing order for three different speeds. J. Orthop. Sports Phys. Ther..

[47] Morse C.I., Thom J.M., Birch K.M., Narici M.V. (2005). Changes in triceps surae muscle architecture with sarcopenia. Acta Physiol. Scand..

[48] Valour D., Ochala J., Ballay Y., Pousson M. (2003). The influence of ageing on the force-velocity-power characteristics of human elbow flexor muscles. Exp. Gerontol..

